# Performance of ChatGPT and Large Language Models on Medical Licensing Exams Worldwide: A Systematic Review and Network Meta-Analysis With Meta-Regression

**DOI:** 10.7759/cureus.94300

**Published:** 2025-10-10

**Authors:** Alousious Kasagga, Aayam Sapkota, Gichin Changaramkumarath, Jane M Abucha, Mekdes M Wollel, Nethra Somannagari, Malik Y Husami, Kirubel T Hailu, Ecem Kasagga

**Affiliations:** 1 Pathology, Peking University, Beijing, CHN; 2 Pathology, California School of Podiatric Medicine, Oakland, USA; 3 Internal Medicine, Chongqing Medical University, Chongqing, CHN; 4 Medicine, American University of Integrative Sciences, Bridgetown, BRB; 5 Medicine, University of Gondar, Gondar, ETH; 6 Neurology, Gandhi Medical College, Hyderabad, IND; 7 Orthopaedics, University Hospitals of Leicester NHS Trust, Leicester, GBR; 8 Public Health, University College Cork, Cork, IRL; 9 Electrical and Computer Engineering, Marquette University, Milwaukee, USA

**Keywords:** artificial intelligence (ai), chatgpt, exam accuracy, large language models (llms), medical education, medical licensing exam, meta-regression, network meta-analysis, systematic review, united states medical licensing exam (usmle)

## Abstract

Large language models (LLMs) are increasingly being tested on national medical licensing examinations, yet existing research is fragmented across models, exam systems, and languages. This study is the first meta-analysis to systematically assess LLM performance across multiple medical licensing exams and languages using pooled estimates, network meta-analysis, and moderator-aware meta-regression. We synthesized accuracy data from 120 evaluations covering 10 exam systems in nine languages, identified through comprehensive searches of PubMed, Web of Science, and Institute of Electrical and Electronics Engineers (IEEE) Xplore, covering the period from 2021 to June 2025.

The random-effects meta-analysis showed that 13 of 16 models exceeded the 60% passing threshold, with GPT-o1 leading at 95.4%, followed by DeepSeek-R1 (92.0%) and GPT-4o (89.4%). P-score rankings and network meta-analysis confirmed the superior performance of GPT-o1, while GPT-3.5 and LLaMA-13B consistently underperformed. Meta-regression revealed a significant variation in accuracy by model version, exam system, and language, with lower performance in Chinese and Japanese exams and higher performance in German and Peruvian settings. After adjustment for exam and language, GPT-o1 and DeepSeek-R1 achieved similar accuracy, both significantly higher than GPT-4. Model type, exam system, and language explained most of the between-study heterogeneity (R² =88.99%), and sensitivity analyses supported the robustness of the pooled estimates.

Overall, several LLMs now approach or exceed the accuracy required to pass standardized medical licensing exams, supporting their potential role in medical education and decision support.

## Introduction and background

Large language models (LLMs) have evolved rapidly into versatile systems capable of performing complex tasks in both clinical and educational contexts [1,2]. Major model families such as Generative Pre-trained Transformer GPT (GPT-3.5, GPT-4, GPT-4o, GPT-o1), Claude (Anthropic’s Claude family; Claude 1.0, Claude 3 Opus), Gemini (Google DeepMind’s Gemini 1.5 Pro), Bard (Google’s Bard, based on PaLM 2), DeepSeek-R (DeepSeek’s retrieval-augmented model; DeepSeek-R1), and LLaMA (Large Language Model Meta AI; LLaMA-13B, LLaMA-70B) have been tested on national medical licensing examinations across diverse settings. These models are built on large-scale datasets that include biomedical literature, academic sources, and general language corpora. Their capacity to interpret clinical questions, apply structured reasoning, and produce coherent, contextually appropriate answers has led to interest in using standardized medical exams as benchmarks for evaluating LLMs, since such exams provide a consistent, high-stakes framework for determining whether LLMs demonstrate the breadth and depth of knowledge expected of licensed physicians [3-5].

National medical licensing exams are rigorous, standardized tests that measure essential medical knowledge, reasoning, and clinical judgment required for professional practice. Examples include the United States Medical Licensing Examination (USMLE), the Professional and Linguistic Assessments Board (PLAB) exam, the Chinese National Medical Licensing Examination (CNMLE), and the German Medical Licensing Examination (GMLE). These exams determine whether candidates meet the minimum competency for independent medical practice. Because of their uniform structure, strong content validity, and international reach, they serve as reliable benchmarks for evaluating LLM performance [3,6]. Unlike informal case vignettes or handpicked question sets, they use consistently developed, board-approved questions across multiple domains, allowing for objective comparisons of model performance across versions, languages, and regions [7,8].

Despite growing interest, evidence on LLM performance in this context remains fragmented. Existing studies differ widely in the models assessed, the exam systems and languages used, the prompting strategies applied, and the performance metrics reported [1,3]. Some studies evaluate a single model in isolation, while others conduct direct head-to-head comparisons. Inconsistencies in study design, scope, and reporting have limited the ability to draw generalizable conclusions regarding the accuracy of LLMs or their comparative performance [2]. Moreover, while some evaluations use official exam questions or public practice materials, others rely on items of unclear origin or uncertain alignment with licensing standards, which raises concerns about their alignment with licensing standards. Prior meta-analyses have typically focused on a single exam, most often the USMLE, or on one language. To date, no study has combined findings across multiple national licensing examinations and languages within a unified quantitative framework, nor examined how exam context and language influence performance across successive LLM generations.

This study addresses that gap by pooling data from 10 licensing examinations across nine languages and applying both network meta-analysis and moderator-based meta-regression. Our objectives were to (i) estimate the pooled accuracy of different models across settings, (ii) compare model families indirectly and rank them relative to each other, and (iii) examine factors such as model type, exam language, and exam system that might influence performance. We also ran sensitivity analyses and risk-of-bias assessments to evaluate the robustness and credibility of the findings. Through this comprehensive approach, we aimed to characterize the current capabilities of LLMs in medical licensing contexts and to identify which model families demonstrate the most consistent and generalizable performance across languages and exam formats.

## Review

Methodology

Search Strategy 

We systematically searched PubMed, Web of Science, and Institute of Electrical and Electronics Engineers (IEEE) Xplore for peer-reviewed studies published between January 1, 2021, and June 1, 2025. The search strategy combined three main concepts using Boolean operators: (i) large language models (e.g., “ChatGPT,” “GPT-4,” “GPT-3.5,” “Med-PaLM,” “Claude,” “Gemini,” “Bard,” “large language model,” “LLM”), (ii) medical licensing examinations (e.g., “USMLE,” “PLAB,” “CNMLE,” “JMLE,” “GMLE,” and related variants), and (iii) performance-related terms (e.g., “accuracy,” “performance,” “evaluation,” “assessment”). To maximize sensitivity, we used both database-specific controlled vocabulary (such as MeSH terms in PubMed) and free-text keywords. Searches were restricted to English-language, peer-reviewed articles involving human subjects, and we excluded preprints, conference abstracts, and non-peer-reviewed sources. We also manually screened the reference lists of all included studies and relevant reviews to capture additional eligible publications. The search process and reporting followed the Preferred Reporting Items for Systematic Reviews and Meta-Analyses (PRISMA) 2020 guidelines [9].

Eligibility Criteria

We included original peer-reviewed studies that quantitatively evaluated the performance of LLMs on national medical licensing exams. Eligible studies were required to report measurable outcomes such as accuracy, mean scores, or pass/fail classifications. Only English-language articles were considered. We excluded preprints, conference abstracts, editorials, and studies that did not directly assess model performance on standardized medical exams.

Study Selection

All search results were imported into Zotero (Corporation for Digital Scholarship, Vienna, VA, US) for reference management, and duplicates were removed. Two reviewers independently screened titles and abstracts in Microsoft Excel (Microsoft Corp., Redmond, WA, US), then assessed full texts against the eligibility criteria. Disagreements were resolved through discussion with a third reviewer. The study selection process was documented using a PRISMA flowchart. 

Data Extraction

Data was extracted independently by two reviewers using a standardized Excel form. Extracted variables included study author, publication year, country, LLM type, exam type, number and source of test items, evaluation format (e.g., multiple-choice), and reported performance metrics (accuracy, score, or pass/fail outcomes). When available, the presence of a comparator group (such as human participants or question bank benchmarks) was also recorded. Any discrepancies were resolved through discussion or adjudication by a third reviewer. Bing (GPT-4) refers to Microsoft’s GPT-4-based interface, which incorporates retrieval and safety features. When reported separately by primary studies, Bing results were analyzed as a distinct variant, and no data were double-counted.

Risk of Bias Assessment

Risk of bias was independently assessed by two reviewers using a modified version of the Quality Assessment of Diagnostic Accuracy Studies - version 2 (QUADAS-2) tool, adapted for studies evaluating AI-based models [10]. The assessment covered four domains: question source quality (representativeness of exam items), LLM use and prompting (clarity and appropriateness of prompting strategies), ground truth answers (validity and consistency of the reference standard), and evaluation consistency (whether all model versions were tested on the same question set under similar conditions). Disagreements were resolved through discussion. Results were summarized and visualized using traffic light plots generated with the robvis R package [11].

Outcome Measures

The primary outcome was model accuracy, defined as the percentage of correctly answered questions on medical licensing examinations. Accuracy values were extracted directly from each study and standardized to a percentage scale for comparability. Secondary analyses investigated differences in performance across exam languages, exam types, and LLM versions, using mixed-effects meta-regression.

Data Synthesis and Statistical Analysis

We synthesized results using a random-effects meta-analysis to estimate the pooled accuracy of LLMs across all eligible studies. To assess the robustness of this estimate, we performed a leave-one-out sensitivity analysis by iteratively removing each study and recalculating the pooled value.

The relative performance of LLMs was assessed using network meta-analysis (NMA), which enabled indirect comparisons among models not directly evaluated in the same study. Relative rankings were generated using P-scores.

To explore heterogeneity, we conducted two mixed-effects meta-regression models: one examining the effect of exam language (e.g., English, Chinese, German) and another examining exam system or region (e.g., USMLE, CNMLE, JMLE). Both models adjusted for variation across LLM versions, using GPT-4 (English, USMLE) as the reference category. For each moderator, we report regression coefficients (β), 95% confidence intervals, p-values, and residual heterogeneity (I²).

All analyses were carried out in R (version 4.5.0) using the metafor package for standard and meta-regression analyses and the netmeta package for NMA [12,13]. Outputs included forest plots, network diagrams, and risk-of-bias visualizations.

Results

Study Selection

**Figure 1:**
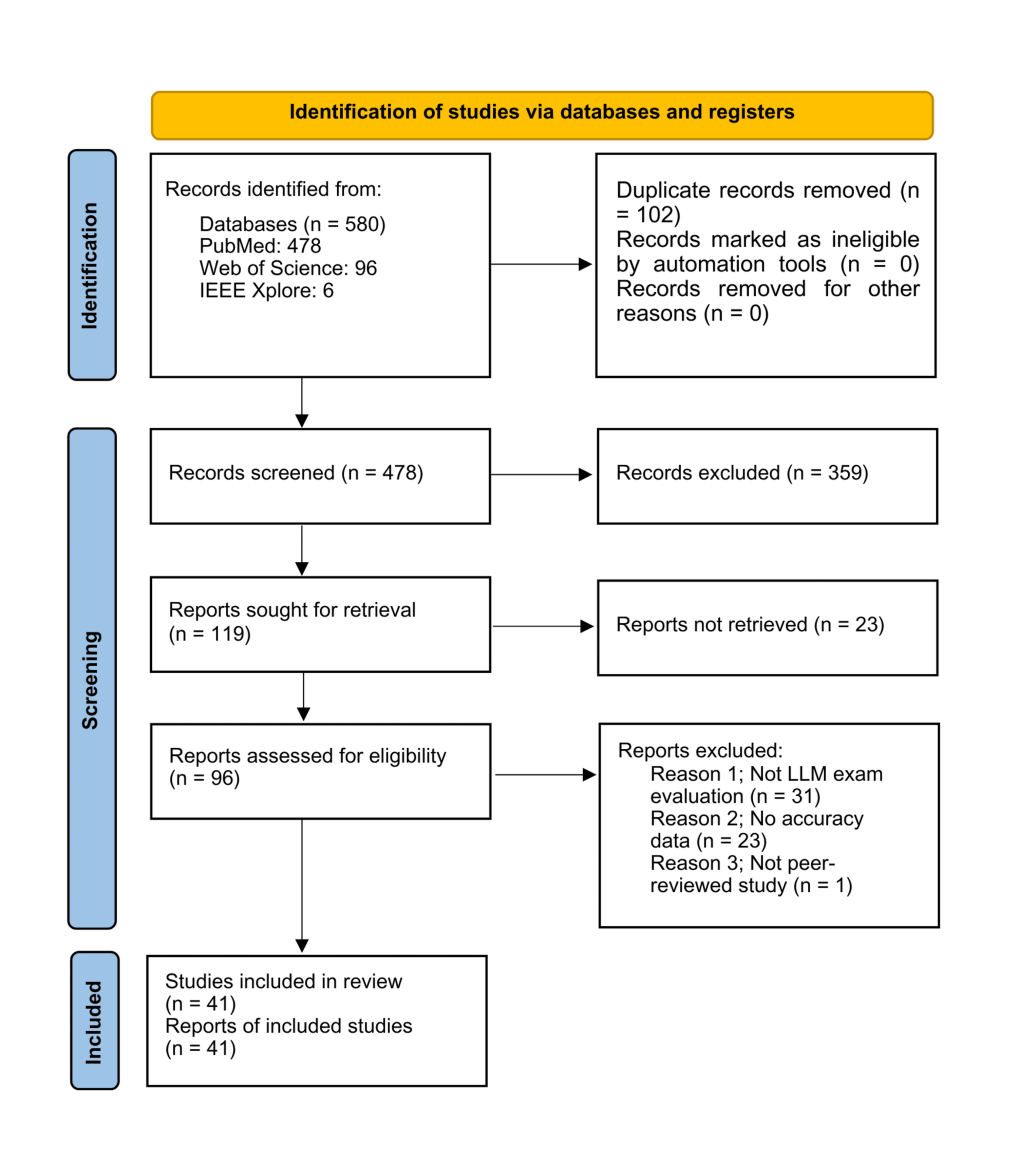
PRISMA flowchart of the study selection process PRISMA: Preferred Reporting Items for Systematic Reviews and Meta-Analyses; LLM: Large Language Model

We identified 580 records across three databases: PubMed (n=478), Web of Science (n=96), and IEEE Xplore (n=6). After removing 102 duplicates, 478 records remained for title and abstract screening. Of these, 359 were excluded. We then retrieved 119 full-text reports, but 23 were inaccessible. Among the 96 full-text articles assessed for eligibility, 55 were excluded for the following reasons: 31 were not evaluations of LLM performance on medical licensing examinations, 23 did not report accuracy data, and one was not peer-reviewed. Ultimately, 41 studies [14-54] met the eligibility criteria and were included in the final review. The study selection process is shown in the PRISMA flow diagram in Figure 1.

Table of Study Characteristics

Table 1 presents the main characteristics of the included studies, including study authors, exam systems and countries, exam languages, LLMs evaluated, the number of questions administered per model, prompting strategies, and reported performance outcomes.

**Table 1:** Characteristics of included studies SMLE: Saudi Medical Licensing Examination; EUNACOM: Examen Único Nacional de Conocimientos de Medicina (Chile); USMLE: United States Medical Licensing Examination; UKMLE: United Kingdom Medical Licensing Examination; CNMLE: Chinese National Medical Licensing Examination; HKMLE: Hong Kong Medical Licensing Examination; IMLE: Iranian Medical Licensing Examination; PMLE: Peruvian Medical Licensing Examination; Revalida: Brazilian Medical Revalidation Exam; TMLE: Taiwan Medical Licensing Examination; JMLE: Japanese Medical Licensing Examination; GMLE: German Medical Licensing Examination; BMLE: Belgian Medical Licensing Examination; LEK: Lekarski Egzamin Końcowy (Polish Final Medical Examination); GPT: Generative Pre-trained Transformer (OpenAI); GPT-3.5, GPT-4, GPT-4o, GPT-o1: versions of OpenAI’s GPT model family; GPT-3.5-Turbo: optimized GPT-3.5 variant; GPT-4V: GPT-4 with vision capabilities; Bard (PaLM 2): Google’s Bard model based on PaLM 2; Gemini 1.5 Pro: Google DeepMind’s multimodal model; Claude 1.0, Claude 1.0+, Claude 3 Opus: Anthropic’s large language models; LLaMA-13B, LLaMA-70B, LLaMA-405B: Meta’s large language models with 13B, 70B, and 405B parameters; DeepSeek-R1: DeepSeek’s retrieval-augmented LLM; Bing (GPT-4): Microsoft Bing chatbot powered by GPT-4. The column "N Questions" shows the number of unique questions each model answered. Ranges indicate multiple item sets, while notations such as "×n exams" reflect the repeated assessments across different exam years or versions.

Study (Author, Year)	Country/Exam	Language	LLMs evaluated	N Questions	Prompting style	Outcome reported
Aljindan et al., 2023 [14]	Saudi Arabia/SMLE	English	GPT-4	220	Zero-shot	Accuracy %, Pass rate
Ebrahimian et al., 2023 [15]	Iran/IMLE	English	GPT-4	200	Zero-shot	Accuracy %, Pass rate
Fang et al., 2023 [16]	China/CNMLE	Chinese	GPT-4	600	Zero-shot	Accuracy %, Pass rate
Flores-Cohaila et al., 2023 [17]	Peru/PMLE	Spanish	GPT-3.5, GPT-4	180	Zero-shot	Accuracy %, Pass rate
Gilson et al., 2023 [18]	USA/USMLE	English	GPT-3.5-Turbo	87-102	Zero-shot	Accuracy %, Pass rate
Gobira et al., 2023 [19]	Brazil/Revalida	Portuguese	GPT-4	81	Zero-shot	Accuracy %, Pass rate
Kung et al., 2023 [20]	USA/USMLE	English	GPT-3.5	336	Zero-shot	Accuracy %, Pass rate
Lai et al., 2023 [21]	UK/UKMLE	English	GPT-4	191	Zero-shot	Accuracy %, Pass rate
Roos et al., 2023 [22]	Germany/GMLE	German	Bing (GPT-4), GPT-3.5-Turbo, GPT-4	590	Zero-shot	Accuracy %, Pass rate
Rosoł et al., 2023 [23]	Poland/LEK	Polish	GPT-3.5, GPT-4	194-196 (×3 exams)	Zero-shot	Accuracy %, Pass rate
Takagi et al., 2023 [24]	Japan/JMLE	Japanese	GPT-3.5, GPT-4	254	Zero-shot	Accuracy %, Pass rate
Tong et al., 2023 [25]	China/CNMLE	Chinese	GPT-4	160	One-shot, explanation requested	Accuracy %, Pass rate
Torres-Zegarra et al., 2023 [26]	Peru/PMLE	Spanish	Bard (PaLM 2), Bing (GPT-4), Claude 1.0 Instant, GPT-3.5, GPT-4	180	Zero-shot	Accuracy %, Pass rate
Wang et al., 2023 [27]	China/CNMLE	Chinese	GPT-3.5	600 (×3 exams)	Zero-shot	Accuracy %, Pass rate
Yanagita et al., 2023 [28]	Japan/JMLE	Japanese	GPT-3.5, GPT-4	292	Zero-shot	Accuracy %, Pass rate
Bicknell et al., 2024 [29]	USA/USMLE	English	GPT-3.5, GPT-4, GPT-4o	750	Zero-shot	Accuracy %, Pass rate
Chen et al., 2024 [30]	China/CNMLE; Hong Kong/HKMLE; UK/UKMLE; USA/USMLE	Chinese, English	Bard (PaLM 2), GPT-3.5, GPT-4, GPT-4o	30-375	Zero-shot	Accuracy %, Pass rate
Garabet et al., 2024 [31]	USA/USMLE	English	GPT-4	1300	Zero-shot	Accuracy %, Pass rate
Huang et al., 2024 [32]	Taiwan/TMLE	Traditional Chinese	GPT-4	200	Zero-shot	Accuracy %, Pass rate
Lin et al., 2024 [33]	Taiwan/TMLE	Traditional Chinese	GPT-4	320	Zero-shot	Accuracy %, Pass rate
Meyer et al., 2024 [34]	Germany/GMLE	German	GPT-3.5, GPT-4	937	Zero-shot	Accuracy %, Pass rate
Mihalache et al., 2024 [35]	USA/USMLE	English	GPT-4	93-120	Zero-shot	Accuracy %, Pass rate
Ming et al., 2024 [36]	China/CNMLE	Chinese	GPT-3.5, GPT-4	600	Zero-shot	Accuracy %, Pass rate
Morreel et al., 2024 [37]	Belgium/BMLE	English	Bard (PaLM 2), Bing (GPT-4), Claude 1.0 Instant, Claude 1.0+, GPT-3.5, GPT-4	95	Zero-shot	Accuracy %, Pass rate
Nakao et al., 2024 [38]	Japan/JMLE	Japanese	GPT-4V	108	Zero-shot	Accuracy %, Pass rate
Rojas et al., 2024 [39]	Chile/EUNACOM	Spanish	GPT-3.5, GPT-4, GPT-4V	540	Zero-shot	Accuracy %, Pass rate
Samuel et al., 2024 [40]	USA/USMLE	English	GPT-3.5, GPT-4	1273	Zero-shot	Accuracy %, Pass rate
Shieh et al., 2024 [41]	USA/USMLE	English	GPT-3.5, GPT-4	109	Zero-shot	Accuracy %, Pass rate
Zong et al., 2024 [42]	China/CNMLE	Chinese	GPT-3.5	597-600 (×5 exams)	Zero-shot	Accuracy %, Pass rate
Altermatt et al., 2025 [43]	Chile/EUNACOM	Spanish	GPT-4o	1062	Zero-shot	Accuracy %, Pass rate
Casals-Farre et al., 2025 [44]	UK/UKMLE	English	GPT-4	191	Zero-shot	Accuracy %, Pass rate
Elkin et al., 2025 [45]	USA/USMLE	English	LLaMA-13B, LLaMA-70B, LLaMA-405B	313	Zero-shot	Accuracy %, Pass rate
Kawahara et al., 2025 [46]	Japan/JMLE	Japanese	GPT-4	2436	Zero-shot	Accuracy %, Pass rate
Kim et al., 2025 [47]	USA/USMLE	English	GPT-3.5, GPT-4, GPT-4o, GPT-o1	325	Zero-shot	Accuracy %, Pass rate
Liu et al., 2025 [48]	Japan/JMLE	Japanese	Claude 3 Opus, Gemini 1.5 Pro, GPT-4, GPT-4o	790	Zero-shot	Accuracy %, Pass rate
Luo et al., 2025 [49]	China/CNMLE	Chinese	GPT-3.5, GPT-4, GPT-4o	600 (×2 exams)	Zero-shot	Accuracy %, Pass rate
Madrid et al., 2025 [50]	Germany/GMLE	German	GPT-3.5, GPT-4	541	Zero-shot	Accuracy %, Pass rate
Tordjman et al., 2025 [51]	USA/USMLE	English	DeepSeek-R1, GPT-o1, LLaMA-405B	323	Zero-shot	Accuracy %, Pass rate
Wu et al., 2025 [52]	Taiwan/TMLE	Traditional Chinese	GPT-3.5, GPT-4, GPT-4o	1188	Zero-shot	Accuracy %, Pass rate
Wu J et al., 2025 [53]	China/CNMLE	Chinese	DeepSeek-R1, GPT-4o	600	Zero-shot	Accuracy %, Pass rate
Yang et al., 2025 [54]	USA/USMLE	English	GPT-3.5-Turbo, GPT-4, GPT-4V	376	Zero-shot	Accuracy %, Pass rate

Risk of Bias Assessment

**Figure 2:**
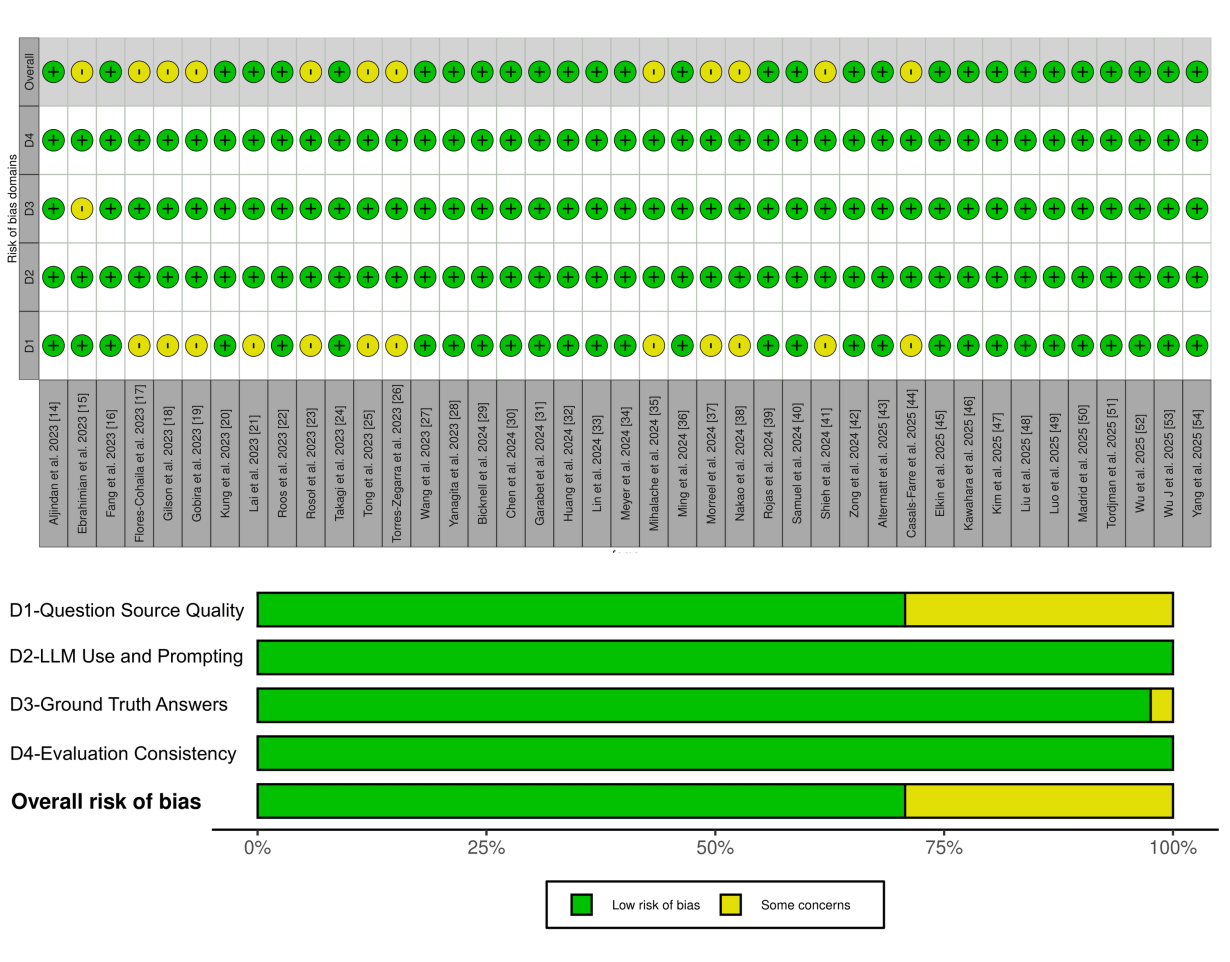
Risk of bias assessment across all included studies LLM: Large Language Model

Most studies were judged to have a low risk of bias across the four QUADAS-2 domains. In Domain 1 (question source quality), 29 studies (71%) were rated low risk, while 12 (29%) had some concerns due to small sample sizes (<200 items). In Domain 2 (LLM use and prompting) and Domain 4 (evaluation consistency), all 41 studies (100%) were judged low risk. In Domain 3 (ground truth answers), 40 studies (98%) were low risk, and one (2%) had some concerns because exam questions were translated from Persian into English for model evaluation. No study was judged to be at high risk of bias. A summary of the assessments is presented in Figure 2 using a traffic light plot.

Pooled Accuracy of Large Language Models

Pooled accuracy varied widely across LLMs. The highest accuracy was observed with GPT-o1 at 95.4% (95% confidence interval [CI]: 93.8-97.0; k=2), followed by DeepSeek-R1 at 92.0% (95% CI: 90.2-93.8; k=2) and GPT-4o at 89.4% (95% CI: 87.2-91.6; k=12). Other high-performing models included Bing (GPT-4) at 84.5% (95% CI: 76.3-92.6; k=3) and LLaMA-405B at 83.9% (95% CI: 81.0-86.7; k=2).

Mainstream GPT-4 showed a pooled accuracy of 82.7% (95% CI: 80.6-84.9; k=45). Models evaluated in only a single study, such as Claude 3 Opus and LLaMA-70B, also performed well, reporting at 82.0% (95% CI: 79.3-84.7; k=1) and 82.4% (95% CI: 78.2-86.6; k=1), respectively. By contrast, older or smaller models showed substantially lower accuracy, including GPT-3.5 at 56.3% (95% CI: 53.1-59.5; k=32) and LLaMA-13B at 44.1% (95% CI: 38.6-49.6; k=1).

**Figure 3:**
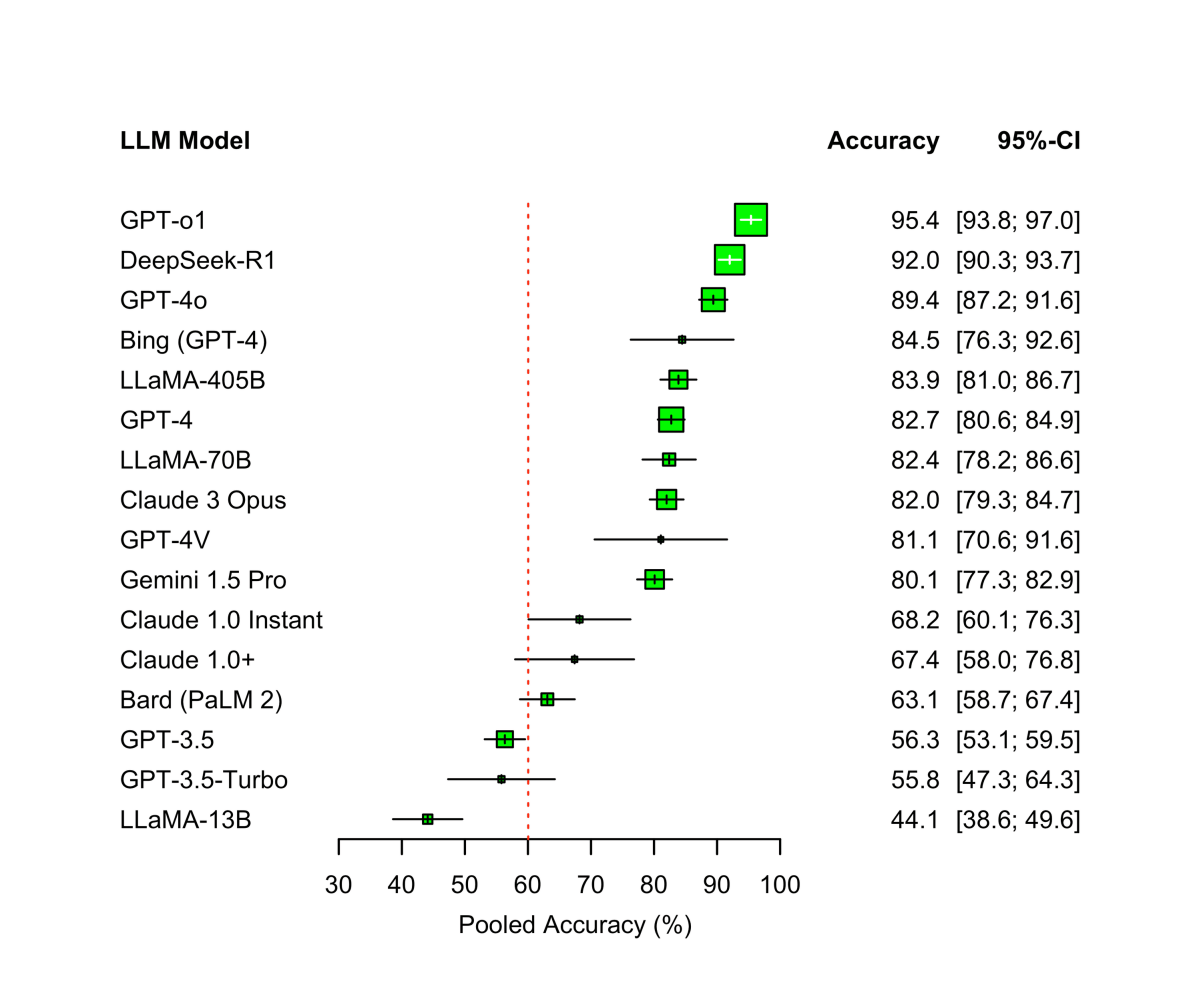
Pooled accuracy of large language models on medical licensing examinations LLM: Large Language Model; CI: Confidence Interval; GPT: Generative Pre-trained Transformer (OpenAI); GPT-3.5, GPT-4, GPT-4o, GPT-o1: versions of OpenAI’s GPT model family; GPT-3.5-Turbo: optimized GPT-3.5 variant; GPT-4V: GPT-4 with vision capabilities; Bard (PaLM 2): Google’s Bard model based on PaLM 2; Gemini 1.5 Pro: Google DeepMind’s multimodal model; Claude 1.0, Claude 1.0+, Claude 3 Opus: Anthropic’s large language models; LLaMA-13B, LLaMA-70B, LLaMA-405B: Meta’s large language models with 13B, 70B, and 405B parameters; DeepSeek-R1: DeepSeek’s retrieval-augmented LLM; Bing (GPT-4): Microsoft Bing chatbot powered by GPT-4. The dashed red line marks the 60% threshold, with higher values indicating greater accuracy.

Using a 60% accuracy threshold as a reference for minimum passing performance, 13 of the 16 models exceeded this benchmark. The three exceptions were GPT-3.5, GPT-3.5-Turbo, and LLaMA-13B. Figure 3 displays the pooled accuracy (%) for each LLM, derived from random-effects meta-analysis with 95% confidence intervals.

Sensitivity Analysis

The leave-one-out sensitivity analysis showed the robustness of the pooled accuracy estimate. Removing any single study changed the pooled accuracy only slightly, with estimates ranging from 73.69% to 74.21% (a span of 0.53 percentage points). The most influential study altered the pooled result by +0.33 percentage points, confirming all shifts were minor and within the CI. These results confirm that the findings are not dependent on any single study and remain robust across the dataset.

Network Diagram of Large Language Models Evaluated on Medical Licensing Examinations

**Figure 4:**
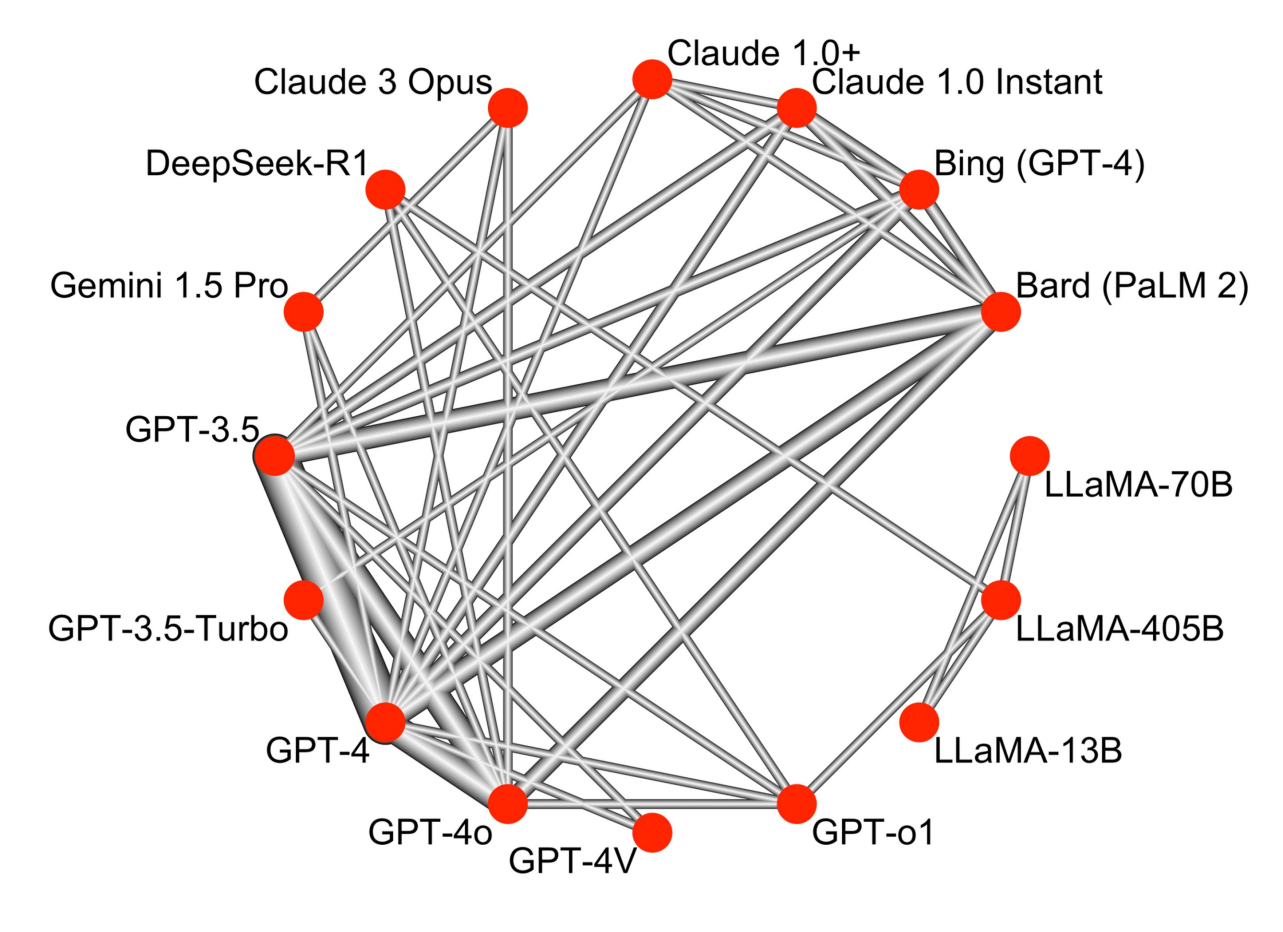
Network diagram of large language models evaluated on medical licensing examinations GPT: Generative Pre-trained Transformer (OpenAI); GPT-3.5, GPT-4, GPT-4o, GPT-o1: Versions of OpenAI’s GPT model family; GPT-3.5-Turbo: Optimized GPT-3.5 variant; GPT-4V: GPT-4 with vision capabilities; Bard (PaLM 2): Google’s Bard model based on PaLM 2; Gemini 1.5 Pro: Google DeepMind’s multimodal model; Claude 1.0, Claude 1.0+, Claude 3 Opus: Anthropic’s large language models; LLaMA-13B, LLaMA-70B, LLaMA-405B: Meta’s large language models (13B, 70B, and 405B parameter versions); DeepSeek-R1: DeepSeek’s retrieval-augmented LLM; Bing (GPT-4): Microsoft Bing chatbot powered by GPT-4

To illustrate comparative relationships across studies, we constructed a network diagram, as shown in Figure 4.

Each node represents an LLM, and each edge denotes a direct head-to-head comparison. Edge thickness reflects the number of studies evaluating a given model pair. The network demonstrated strong interconnectivity, particularly around mainstream models. GPT-4 was the most connected node, directly compared with 11 other models across 45 studies (11 connections; k=45). GPT-3.5 followed closely, connected to seven models (k=32), including frequent comparisons with GPT-4 (k=21), Bard (PaLM 2) (k=6), and GPT-4o (k=8). Other well-connected models included Bard (six connections; k=6), GPT-4o (five connections; k=12), and GPT-o1 (four connections; k=2). In contrast, peripheral models such as LLaMA-405B (one connection; k=2), LLaMA-70B (one connection; k=1), and Claude 1.0+ (one connection; k=1) had fewer direct comparisons but were indirectly linked through the broader network. Overall, the network displayed a robust structure anchored by mainstream models, supporting both direct and indirect comparisons across the full set of LLMs.

Network Meta-analysis of Large Language Model Accuracy

We conducted a network meta-analysis to compare the accuracy of LLMs with GPT-4 as the reference comparator. GPT-o1 demonstrated the highest relative accuracy, with a mean difference (MD) of 12.8% (95% CI: 5.1-20.6) compared to GPT-4. DeepSeek-R1 also showed significantly higher performance (MD: 10.7%, 95% CI: 1.9-19.4), followed by GPT-4o (MD: 6.7%, 95% CI: 3.6-9.8). 

Several models, including Claude 3 Opus, LLaMA-405B, Gemini 1.5 Pro, GPT-4V, and LLaMA-70 B, did not differ significantly from GPT-4. In contrast, multiple models performed significantly worse than GPT-4, including Claude 1.0+ (MD: -15.3%, 95% CI: -23.9 to -6.6), Claude 1.0 Instant (-19.0%, 95% CI: -25.3 to -12.7), GPT-3.5 (-22.6%, 95% CI: -24.8 to -20.4), Bard (PaLM 2) (-23.5%, 95% CI: -27.4 to -19.6), GPT-3.5-Turbo (-24.8%, 95% CI: -34.5 to -15.2), and LLaMA-13B (-39.3%, 95% CI: -55.1 to -23.6).

**Figure 5:**
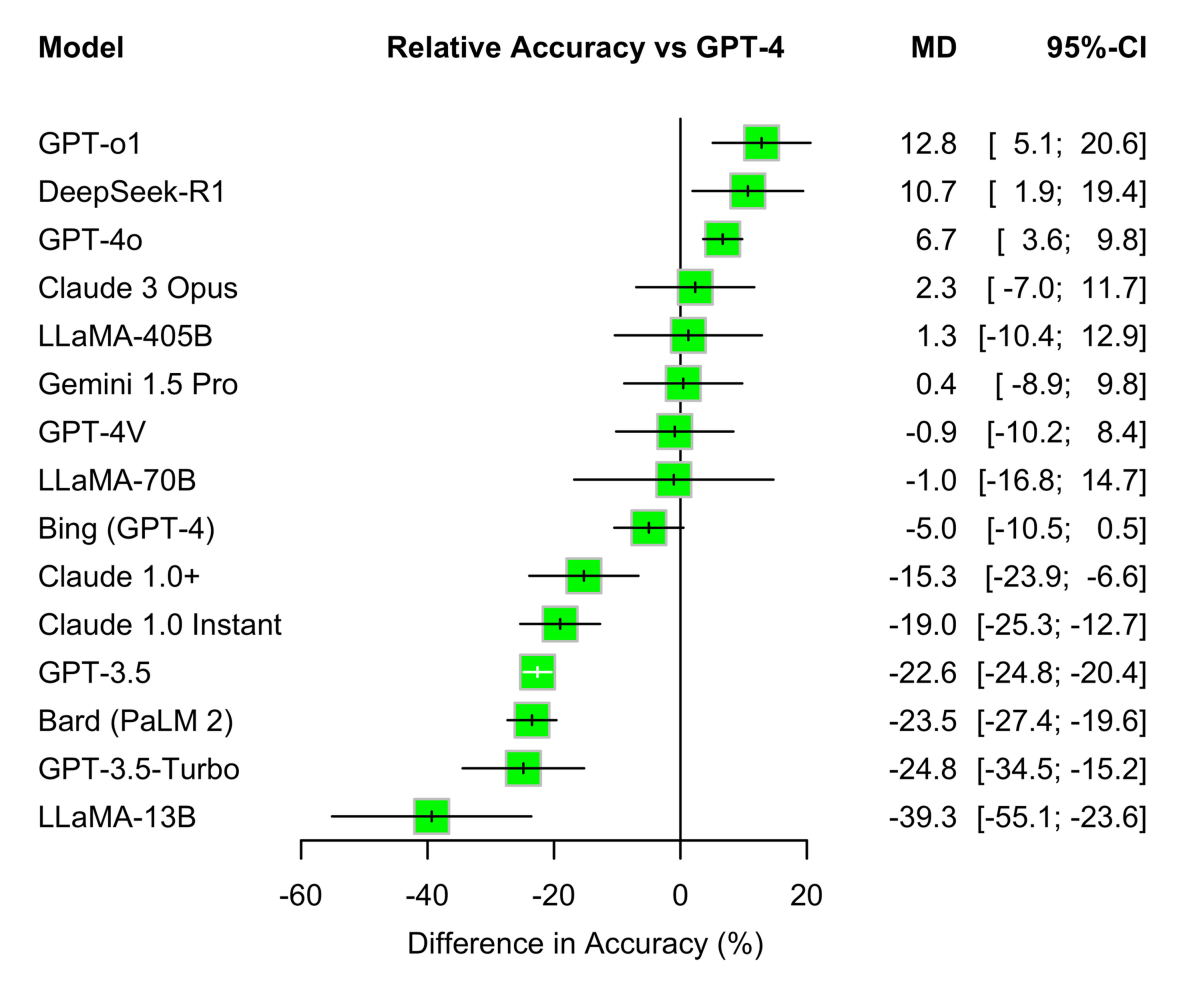
Network meta-analysis of large language model accuracy MD: Mean Difference; CI: Confidence Interval; GPT: Generative Pre-trained Transformer (OpenAI); GPT-3.5, GPT-4, GPT-4o, GPT-o1: Versions of OpenAI’s GPT model family; GPT-3.5-Turbo: Optimized GPT-3.5 variant; GPT-4V: GPT-4 with vision capabilities; Bard (PaLM 2): Google’s Bard model based on PaLM 2; Gemini 1.5 Pro: Google DeepMind’s multimodal model; Claude 1.0, Claude 1.0+, Claude 3 Opus: Anthropic’s large language models; LLaMA-13B, LLaMA-70B, LLaMA-405B: Meta’s large language models (13B, 70B, and 405B parameter versions); DeepSeek-R1: DeepSeek’s retrieval-augmented LLM; Bing (GPT-4): Microsoft Bing chatbot powered by GPT-4. Confidence intervals are shown for each comparison. Positive values indicate higher accuracy than GPT-4, while negative values reflect lower accuracy.

These findings showed that newer models, such as GPT-o1, DeepSeek-R1, and GPT-4o, outperformed GPT-4, whereas earlier or smaller models, particularly the GPT-3.5 variants and LLaMA-13B, consistently underperformed. Figure 5 presents the relative accuracy of each LLM compared with GPT-4, based on network meta-analysis.

P-score Rankings of Large Language Model Accuracy

**Figure 6:**
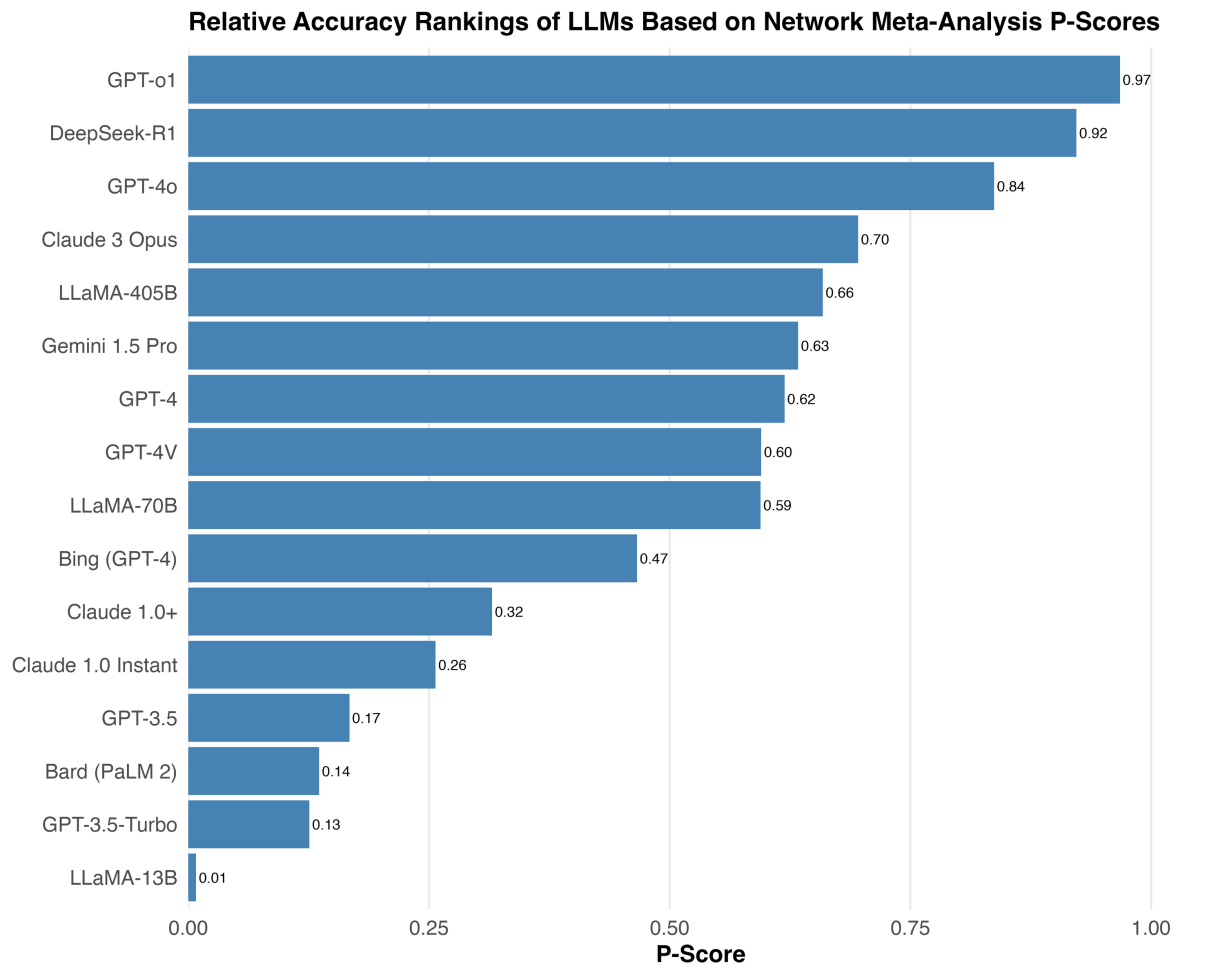
P-score rankings of large language model accuracy from network meta-analysis. LLM: Large Language Model; GPT: Generative Pre-trained Transformer (OpenAI); GPT-3.5, GPT-4, GPT-4o, GPT-o1: versions of OpenAI’s GPT model family; GPT-3.5-Turbo: optimized GPT-3.5 variant; GPT-4V: GPT-4 with vision capabilities; Bard (PaLM 2): Google’s Bard model based on PaLM 2; Gemini 1.5 Pro: Google DeepMind’s multimodal model; Claude 1.0, Claude 1.0+, Claude 3 Opus: Anthropic’s large language models; LLaMA-13B, LLaMA-70B, LLaMA-405B: Meta’s large language models (13B, 70B, and 405B parameter versions); DeepSeek-R1: DeepSeek’s retrieval-augmented LLM; Bing (GPT-4): Microsoft Bing chatbot powered by GPT-4.

The P-score, a probabilistic ranking metric derived from network meta-analysis, reflects the relative likelihood that one model outperforms others. It represents the frequentist analog of the Surface Under the Cumulative Ranking Curve (SUCRA), commonly reported in Bayesian network meta-analyses. Higher values correspond to stronger overall performance. In our analysis, GPT-o1 ranked highest (P-score=0.968), followed by DeepSeek-R1 (0.923) and GPT-4o (0.837), which was consistent with its superior diagnostic reasoning accuracy. At the other end of the spectrum, GPT-3.5 (0.167), Bard PaLM 2 (0.136), and LLaMA-13B (0.008) ranked lowest, indicating weaker and less reliable performance across studies. Figure 6 displays the P-score-based ranking of LLMs derived from the network meta-analysis.

Meta-regression of Large Language Model Accuracy

We conducted a mixed-effects meta-regression to compare accuracy across models, using GPT-4 as the reference comparator. The intercept (β=83.23, 95% CI: 80.60 to 85.86, p<0.0001) represents the estimated average accuracy of GPT-4 across all included studies. Several models performed significantly better than GPT-4, including DeepSeek-R1 (β=12.71, 95% CI: 5.15 to 20.27, p=.0010), GPT-o1 (β=12.12, 95% CI: 4.48 to 19.76, p=0.0019), and GPT-4o (β=9.12, 95% CI: 5.49 to 12.75, p<0.0001). 

By contrast, several models performed significantly worse than GPT-4, such as GPT-3.5 (β=-24.29, 95% CI: -27.07 to -21.51, p<0.0001), GPT-3.5-Turbo (β=-28.26, 95% CI: -33.93 to -22.59, p<0.0001), and LLaMA-13B (β=-39.13, 95% CI: -50.74 to -27.52, p<0.0001). Bard (PaLM 2), Claude 1.0 Instant, and Claude 1.0+ also showed significantly lower accuracy than GPT-4. For other models, including Claude 3 Opus, Gemini 1.5 Pro, GPT-4V, LLaMA-70B, Bing (GPT-4), and LLaMA-405B, accuracy did not differ significantly from GPT-4, as their confidence intervals overlapped with zero. 

**Figure 7:**
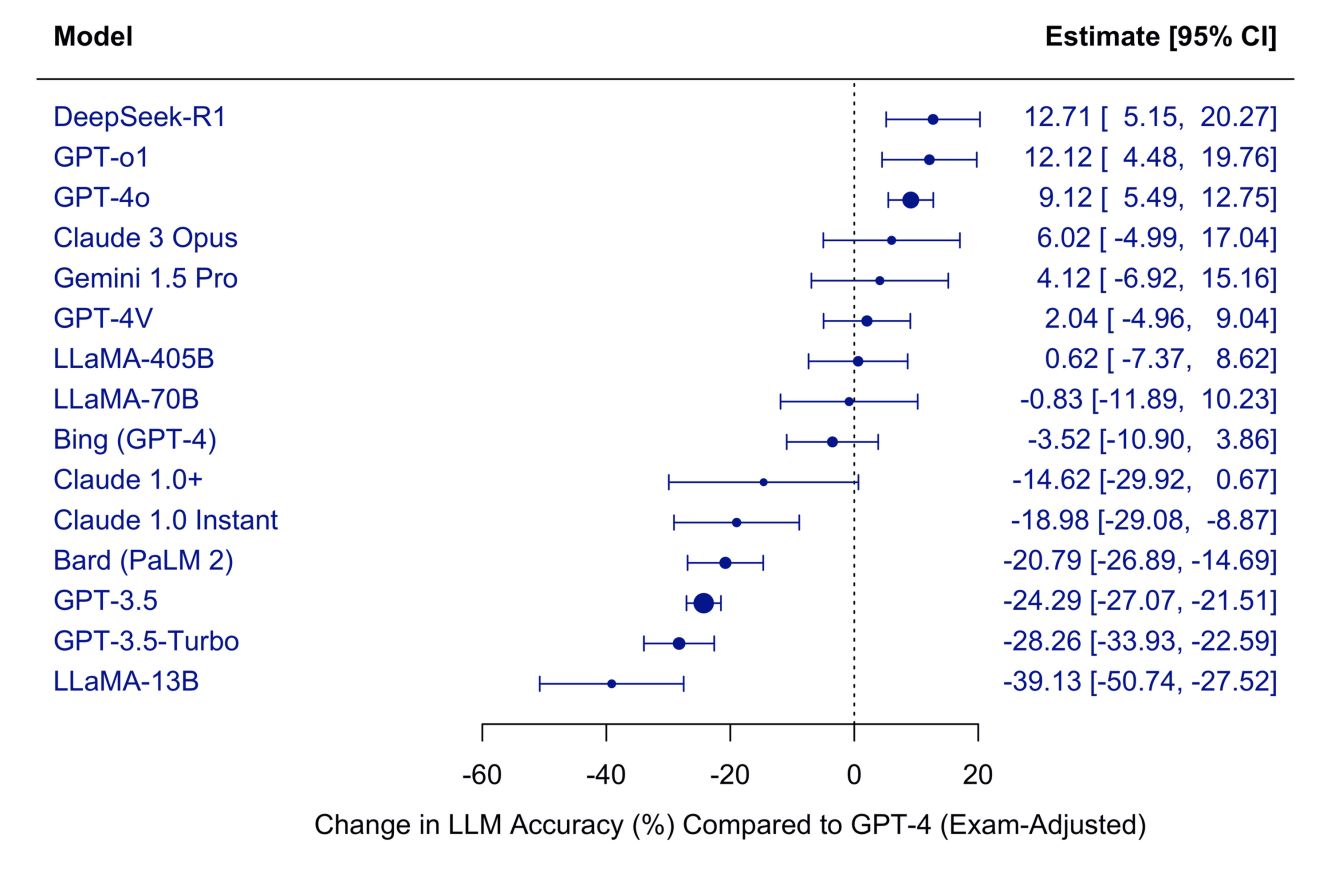
Meta-regression of large language model accuracy relative to GPT-4 LLM: Large Language Model; CI: Confidence Interval; GPT: Generative Pre-trained Transformer (OpenAI); GPT-3.5, GPT-4, GPT-4o, GPT-o1: versions of OpenAI’s GPT model family; GPT-3.5-Turbo: optimized GPT-3.5 variant; GPT-4V: GPT-4 with vision capabilities; Bard (PaLM 2): Google’s Bard model based on PaLM 2; Gemini 1.5 Pro: Google DeepMind’s multimodal model; Claude 1.0, Claude 1.0+, Claude 3 Opus: Anthropic’s large language models; LLaMA-13B, LLaMA-70B, LLaMA-405B: Meta’s large language models (13B, 70B, and 405B parameter versions); DeepSeek-R1: DeepSeek’s retrieval-augmented LLM; Bing (GPT-4): Microsoft Bing chatbot powered by GPT-4. Positive values indicate higher accuracy than GPT-4, whereas negative values indicate lower accuracy.

Overall, the analysis revealed substantial heterogeneity in model performance. Newer models such as GPT-4o, GPT-o1, and DeepSeek-R1 surpassed GPT-4, while earlier-generation models, particularly GPT-3.5 and LLaMA-13B, lagged behind. Figure 7 illustrates the estimated change in accuracy (%) for each LLM relative to GPT-4, adjusted for exam type.

Meta-regression of Multilingual Performance of Large Language Models

We conducted a mixed-effects meta-regression to assess how examination language influenced LLM performance, with both model type and language included as moderators. GPT-4 evaluated in English served as the reference group, and its performance was represented by the intercept (β=83.40, p<0.0001). This served as the baseline for all other models and language effects.

The analysis included 120 effect sizes and accounted for a large proportion of between-study heterogeneity (R²=86.93%). However, residual heterogeneity remained considerable (I²=89.15%), suggesting the presence of additional unexplained variability. The overall test of moderators was statistically significant (QM(df=22) =659.52, p<0.0001).

Relative to English, the performance was significantly lower in Chinese (β=-7.97, p<0.0001) and Japanese (β=-7.20, p=0.0025), while it was significantly higher in German (β=+6.71, p=0.0060). Other languages, including Spanish, Polish, and Traditional Chinese, did not differ significantly from English.

**Figure 8:**
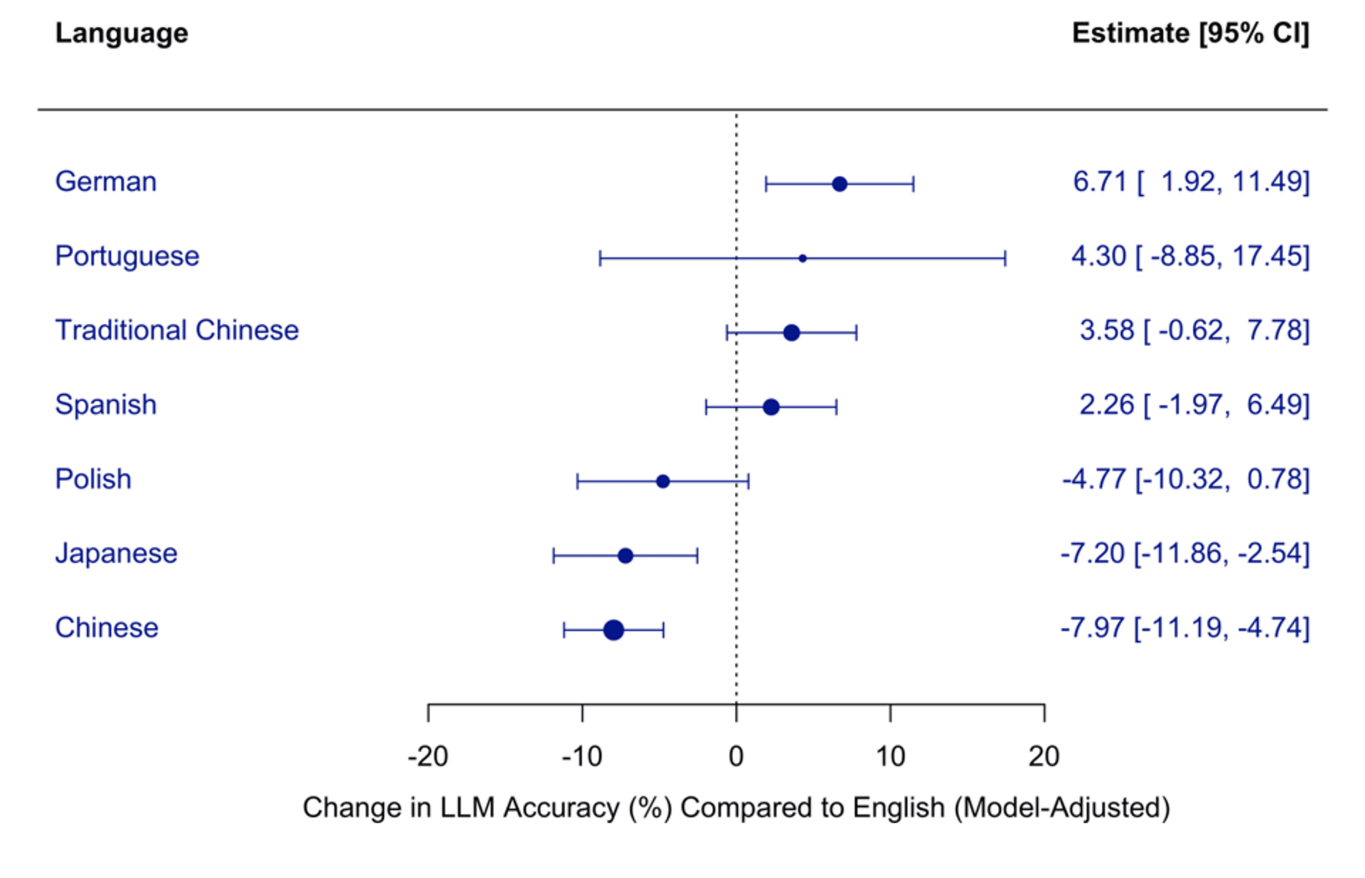
Meta-regression of large language model accuracy by examination language LLM: Large Language Model; CI: Confidence Interval Estimates are presented with 95% confidence intervals; positive values indicate higher accuracy than English, and negative values indicate lower accuracy.

These results highlight an important variation in multilingual LLM performance. Languages such as German may benefit from stronger alignment or richer representation in pretraining data, whereas languages like Chinese and Japanese may pose greater challenges due to the complexity of tokenization, linguistic structure, or limited representation in training corpora. Figure 8 shows the estimated change in accuracy (%) across languages compared with English, adjusted for model type.

Meta-regression of Large Language Model Accuracy Across Medical Licensing Examinations

To examine whether LLM performance varied across different medical licensing exams, we conducted a mixed-effects meta-regression, including both model type and exam system as moderators. GPT-4 evaluated on the USMLE served as the reference group, estimated by the model intercept (β=83.23, p<0.0001).

The analysis incorporated 120 effect sizes and explained a substantial proportion of between-study heterogeneity (R²=88.99%). However, substantial residual heterogeneity remained (I²=87.69%), suggesting considerable unexplained variability. The overall test of moderators was statistically significant (QM(df=28)=780.93, p<0.0001).

Relative to the USMLE, the performance was significantly lower on the CNMLE (β=-7.74, p<0.0001), JMLE (β=-7.26, p=0.0016), and IMLE (β=-14.73, p=0.0169). In contrast, it was significantly higher on the PMLE (β=+7.46, p=0.0063) and GMLE (β=+7.09, p=0.0026). Accuracy on other examinations, including those from the UK, Taiwan, Chile, Poland, and Belgium, did not differ significantly from the US benchmark.

**Figure 9:**
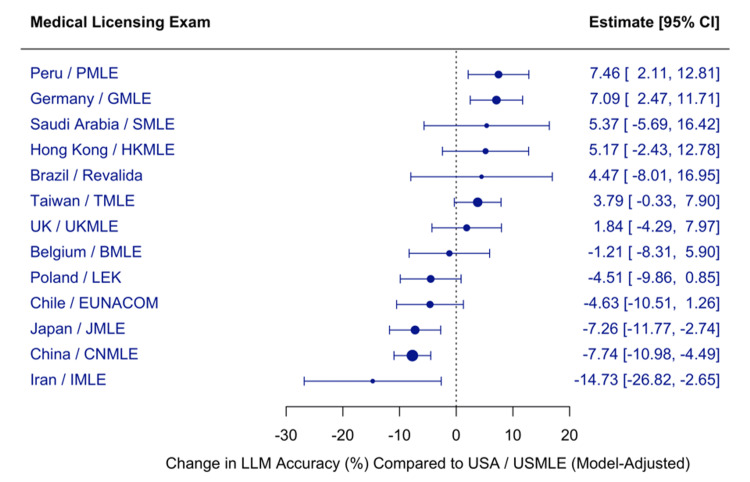
Meta-regression of large language model accuracy across medical licensing examinations LLM: Large Language Model; CI: Confidence Interval; USMLE: United States Medical Licensing Examination; PMLE: Peruvian Medical Licensing Examination; GMLE: German Medical Licensing Examination; SMLE: Saudi Medical Licensing Examination; HKMLE: Hong Kong Medical Licensing Examination; Revalida: Brazilian Medical Revalidation Exam; TMLE: Taiwan Medical Licensing Examination; UKMLE: United Kingdom Medical Licensing Examination; BMLE: Belgian Medical Licensing Examination; LEK: Lekarski Egzamin Końcowy (Polish Final Medical Examination); EUNACOM: Examen Único Nacional de Conocimientos de Medicina (Chile); JMLE: Japanese Medical Licensing Examination; CNMLE: Chinese National Medical Licensing Examination; IMLE: Iranian Medical Licensing Examination. Values are shown with 95% confidence intervals; positive estimates indicate higher accuracy than the USMLE, and negative estimates indicate lower accuracy.

Figure 9 presents the estimated change in accuracy (%) across medical licensing exams compared with the USMLE, adjusted for model type.

Discussions

Overview of Key Findings

This study systematically evaluated the performance of large language models across multiple medical licensing examinations using pooled meta-analysis and meta-regression approaches. Overall, 13 of the 16 models achieved a pooled accuracy estimate above the 60% passing threshold, underscoring their growing potential in medical knowledge assessment. In both pooled and network analyses, GPT-o1, DeepSeek-R1, and GPT-4o consistently ranked as the highest-performing models, each demonstrating significantly greater accuracy than GPT-4. By contrast, GPT-3.5, GPT-3.5-Turbo, and LLaMA-13B consistently lagged.

Meta-regression that adjusted for exam type confirmed that DeepSeek-R1, GPT-4o, and GPT-o1 outperformed GPT-4, indicating that their superior performance is consistent across different test formats. Other models, including Gemini 1.5 Pro and members of the Claude family, showed no significant difference from GPT-4, pointing to variability in model reliability. Findings were robust to sensitivity testing, supporting the stability of the pooled estimates.

Impact of the Model Architecture and Versioning

The results revealed clear differences in performance across model generations and architectures. GPT-3.5 and GPT-3.5-Turbo consistently showed the lowest accuracy in pooled, network, and regression analyses, reflecting the limitations of earlier-generation models [29]. In contrast, DeepSeek-R1 achieved the highest model-adjusted accuracy, with GPT-o1 performing at a similar level and both exceeding GPT-4o. Notably, GPT-o1’s performance was closer to that of DeepSeek-R1 than to GPT-4o. Although GPT-4o remained among the top performers, these findings demonstrated that high accuracy is not limited to direct successors of GPT-4. DeepSeek-R1, believed to be refined from the GPT-4 framework through domain adaptation and additional tuning, demonstrated how targeted development can yield superior performance even when models share a common architectural foundation [51].

Impact of the Exam Type

Model performance differed substantially across national medical licensing examinations. Relative to GPT-4’s performance on the USMLE, accuracy was significantly lower on the CNMLE, JMLE, and IMLE. These differences may reflect challenges such as translation complexity, variations in exam structure and content emphasis, or cultural and clinical framing that current LLMs may not process consistently [30,36].

In contrast, accuracy was significantly higher on the PMLE and GMLE. While the underlying drivers remain uncertain, one hypothesis is that these exams may align more closely with pretraining data or employ formats that better suit LLM reasoning. These observations should be considered hypothesis-generating and require further validation. Performance on exams from the UK, Taiwan, Chile, Poland, and Belgium did not differ significantly from the USMLE, suggesting a degree of generalizability across regions.

Although the exam system explained a large proportion of heterogeneity, considerable unexplained variance remained, indicating that examination type is only one factor shaping LLM performance. These findings underscore the importance of considering exam context when interpreting and comparing model accuracy [30].

Impact of the Exam Language

The exam language significantly moderated model performance in uneven but interpretable ways. After adjustment for model type, we found that accuracy was notably lower for Chinese and Japanese exams compared to English. These shortcomings likely stemmed from a mix of factors, including the complexity of tokenization, the underrepresentation of non-Latin scripts in pretraining datasets, and the specialized clinical terminology used in East Asian assessments [3,55]. While the reasons for this difference remained unclear, one hypothesis is that German exam content may benefit from a closer lexical connection to English medical terminology or a stronger representation in training data. These observations are hypothesis-generating and highlight the need for further cross-linguistic validation. For Spanish, Polish, and Traditional Chinese, no significant differences were noted, suggesting that performance can reach levels comparable to English when the target language is well-represented or when the exam content aligns with standardized medical terminology. 

Overall, these findings highlight that multilingual performance varies significantly across LLMs. Even the best-performing models exhibit measurable accuracy gaps in languages featuring complex scripts or with limited corpus coverage. Addressing these disparities will require targeted multilingual fine-tuning and a broader integration of high-quality clinical texts in underrepresented languages.

Addressing Heterogeneity

Substantial heterogeneity was observed across studies, which is expected given the diversity in model architectures, exam formats, and languages evaluated. The pooled random-effects meta-analysis yielded a high I² value of 98.63%, indicating considerable between-study variability. Much of this variability, however, was explained by meta-regression models. When model type and exam group were included as moderators, the model accounted for 88.99% of the heterogeneity. Similarly, the inclusion of model and language explained 86.93% of the between-study variance. These results suggested that key characteristics at the study level can meaningfully account for performance variation among LLMs.

The robustness of the pooled results was further supported by leave-one-out sensitivity analyses. By sequentially excluding individual studies, only minor adjustments were seen in the pooled accuracy estimate, which fluctuated slightly between 73.69% and 74.21%. This demonstrated that no single study disproportionately influenced the overall findings.

In summary, although high heterogeneity is expected in this rapidly evolving field, it can be systematically addressed through appropriate model adjustments and robust analytical strategies.

Clinical Applications and Future Research

The impressive performance of models like DeepSeek-R1, GPT-o1, and GPT-4o across various medical licensing exams and languages suggested that some LLMs are approaching a level of reliability with potential real-world applications. In medical education, these models could serve as interactive tutors to support exam preparation, reinforce clinical reasoning, or provide study resources in multiple languages [2,20]. In clinical practice, LLMs are being increasingly explored as assistants for tasks such as drafting documentation, summarizing patient records, and retrieving clinical guidelines [2,4]. The high levels of accuracy reported in this meta-analysis further underscore their potential in these applications.

Nevertheless, accuracy on its own is not sufficient to justify clinical deployment. For LLMs to be integrated into standard care, they must undergo thorough evaluations for consistency, safety, and transparency. Key areas of focus should include their performance with real patient data, ability to handle ambiguity, and reliability in rare or high-stakes situations. Moreover, it’s crucial for researchers to consider how these models fit within ethical standards, legal frameworks, and patient expectations [3,4,40].

Looking ahead, future research should emphasize external validation within clinical workflows, assess the long-term impacts on care quality, and develop safeguards to mitigate potential risks. As the capabilities of LLMs continue to evolve, the key question will shift from whether these tools can be used in healthcare to how they can be implemented responsibly.

Strengths and Limitations

This meta-analysis represents the first comprehensive evaluation of LLM performance across a broad spectrum of medical licensing exams and languages, utilizing pooled, network, and meta-regression approaches. One of the main strengths of our study is the wide variety of the research included, which encompasses 120 evaluations across 10 exam systems and nine languages. This diversity allowed us to scrutinize LLMs in different real-world testing scenarios rather than just in isolated or controlled environments. 

Methodologically, we employed a combination of pooled meta-analysis, network meta-analysis, and mixed-effects meta-regression, each providing complementary insights. Pooled estimates summarized overall accuracy within models, network meta-analysis enabled indirect comparisons and ranking where direct evaluations were not available, and meta-regression identified the influence of moderators such as exam type and language. Sensitivity analyses, including leave-one-out testing, further validated the stability of our pooled estimates, while moderator analyses explained much of the observed variability between studies.

However, our study also has its limitations. Despite adjusting for key moderators, residual heterogeneity remained high. This suggested that other factors, such as differences in prompting, question format, or domain-specific content, may have also influenced performance. In some specific exam or language subgroups, the small number of available studies limited the reliability of subgroup comparisons. Although all included studies used multiple-choice questions, variation in question style and difficulty across different exams may have impacted the results. Additionally, several non-English exams were translated into English prior to model evaluation, which could have introduced potential bias if the translation altered question clarity or difficulty. Finally, although accuracy was the focus of this analysis, it does not capture the full spectrum of LLM capabilities. Other dimensions, such as interpretability, consistency, and safety in clinical contexts, remain essential but were beyond the scope of this study.

## Conclusions

This meta-analysis synthesized evidence from 120 evaluations of large language models across 10 medical licensing examinations and nine languages, marking it as the first meta-analysis to establish benchmark evidence for LLM performance across diverse medical licensing contexts. First, we estimated the pooled accuracy of large language models across various settings, demonstrating that their overall performance now approached or exceeded human pass thresholds in many contexts. Second, we compared model families using network meta-analysis and found that GPT-o1, DeepSeek-R1, and GPT-4o ranked highest in overall accuracy. Third, we examined moderators such as model type, exam language, and system, and showed that newer multimodal architectures and English-language exams were associated with superior results.

As LLM development progresses, these high-performing models set a standard for future validation and comparative assessment. The key challenge ahead is not whether LLMs can perform well on medical licensing exams, but how they can be responsibly integrated into healthcare and education, ensuring safety, transparency, and equity in their real-world implementation.
